# Barriers and facilitators for integration of guidelines on operating health shops: a case of family planning services

**DOI:** 10.1186/s40545-021-00337-4

**Published:** 2021-11-16

**Authors:** Joseph M. Zulu, Doreen Sitali, Zubin Cyrus Shroff, Geetanjali Lamba, George Sichone, Charles Michelo, Chileshe H. Mpandamabula, Wesely Mwambazi, Cecilia Mwenda, Malizgani P. Chavula

**Affiliations:** 1 Department of Health Promotion and Education, School of Public Health, University of Zambia, PO Box 50110, Lusaka, Zambia; 2 Department of Health Policy and Management, School of Public Health, University of Zambia, PO Box 50110, Lusaka, Zambia; 3 Alliance for Health Policy and Systems Research, Science Division, World Health Organization, Avenue Appia 20, 1211 Geneva, Switzerland; 4 Participatory Research and Innovations Management, Lusaka, Zambia; 5 School of Public Health, Department of Epidemiology and Biostatistics, University of Zambia, Lusaka, Zambia; 6 Rigor Data Research, Lusaka, Zambia; 7 The Zambia Medicines Regulatory Authority, Lusaka, Zambia

**Keywords:** Health shops, Guidelines, Integration, Health system, Zambia

## Abstract

**Background:**

The Zambia Medicines Regulatory Authority (ZAMRA) piloted the implementation of Guidelines on Operating Health Shops in Zambia in 2016, with a view to making basic medicines more accessible to communities. The guidelines aim to transform ordinary drug shops into health shops, which are dispensing facilities permitted to sell a ZAMRA-prescribed list of medicines over the counter. However, studies that explore the integration and uptake of guidelines into the health system are lacking. This study aims to inform future improved implementation of these guidelines by examining the current acceptability of guidelines within the Zambian health system, especially in relation to family planning services.

**Methodology:**

Data collected through documentary review, key informant interviews with district pharmacists, staff from ZAMRA and in-depth interviews with 24 health shop owners and dispensers were analyzed using thematic analysis. A conceptual framework on the integration of health innovations into health systems guided the analysis.

**Results:**

The Guidelines on Operating Health Shops were implemented to address the problem of inadequate access to quality medicines especially in rural areas. Factors that facilitated the acceptability of the guidelines included their perceived relevance and simplicity, comprehensive training and improved knowledge among health shop operators on the guidelines, development of a governance and reporting structure or steering committee at the national level as well as perceived improved health outcomes at the community level. Factors that hindered acceptability of the guidelines included the high cost of implementing them, a restricted list of drugs which affected consumer choice, limited communication between the local council and the operators of health shops, health shop owners not owning the health shop premises restricting their ability to adapt the building, and cultural norms which constrained uptake of family planning services.

**Conclusion:**

In addition to training, facilitating the acceptability of the guidelines among health shop owners requires paying attention to operational issues such as location, ownership of the shop, size of infrastructure as well as financial costs of implementing guidelines through decentralizing the registration process and thus reducing the cost of registration. It is also important to have effective communication strategies between operators and the regulators of health shops.

## Background

Many low- and middle-income countries (LMICs), including Zambia, still grapple with a high burden of preventable disease compared to high income countries [1]. In order to help reduce this burden, health systems need to be strengthened [2]. One way to strengthen health systems is building capacity of supply chains of essential medications, including family planning [3]. Zambia faces, among other issues, considerable reproductive health challenges. For instance, one in seven female (15%) adolescents aged 15–19 years are married (or in union) compared to only 1% of their male counter parts; 21.5% of married girls aged 15–19 have an unmet need for family planning; the percentage of adolescents who have begun child bearing ranges from 6% among those aged 15 years to 53% among those aged 19 years [4].

In most African countries, many rural populations rely on private drug retailers for point of care treatment for several reasons. These include limited availability of public sector health services and a requirement to travel long distances to government health facilities. Further, drug shops are preferred because of favorable opening hours, being given medicines on demand (perceived service quality) and drug shops and their owners being seen as closer to communities [2, 5, 6]. Thus, there is a need for private stakeholder engagement in the supply chain, including family planning products.

However, several challenges characterize the operations of these drug shops [7]. For example, some shops sell out of date drugs while others sell some drugs without a prescription [7]. Additional challenges include dispensing drugs that are not legally appropriate [7, 8]. Studies further reported that drugs were often sold in loose strips, as opposed to in their original packaging [2, 5, 6]. In addition, very few providers referred customers to other health facilities because they wanted to maintain clients’ loyalty to them [2, 5, 6]. Such challenges have negative implications on health systems performance with regard to delivery of appropriate health care to the people [2, 5, 6]. One strategy of addressing the above challenges is engaging the private sector with specific initiatives that target private drug retailers, to ensure the appropriate dispensing of quality assured drugs [2, 5, 6].

Initiatives for improving service delivery by private drug retailers implemented in Africa include the provision of training and algorithms to staff in drug shops, linking drug shops to established supply chains to ensure medication quality, enhanced regulation and monitoring of supplies and drug dispensing patterns [2, 5, 6]. Therefore, to enhance the supply of medicines in rural Zambia, the Zambia Medicines Regulatory Authority (ZAMRA), a regulatory body reporting to the Ministry of health, developed and piloted the implementation of *Guidelines on Operating Health Shops* in Zambia in 2016. The guidelines aim to transform ordinary drug shops into health shops, and open new health shops [9]. A health shop is a dispensing facility permitted to sell a ZAMRA-prescribed list of medicines, such as anti-malarials and oral contraceptives, over the counter. The health shop initiative aims to increase access to quality medicines, especially in rural areas. The guidelines outline key standards for the health shop such as personnel requirements, safety and security of premises, supply of medicines, roles and responsibilities of health shop owners, hygiene within the premises, record keeping facilities and disposal of pharmaceutical and non-pharmaceutical waste. These guidelines were piloted in Chinsali and Nakonde Districts in Muchinga Province and Mongu and Senanga Districts of Western province of Zambia.

However, studies that explore the facilitators and barriers to acceptability and adoption of the guidelines, as well as potential solutions or strategies to address implementation challenges are lacking. This study thus aimed to explore the factors that influence the acceptability of guidelines within the Zambian Health System, especially in relation to family planning services. It also aimed to understand how the implementation of the guidelines contributed to a stronger health system as well as draw the lessons to inform improved future implementation.

### Conceptual framework

A conceptual framework on the integration of health innovations into health systems by Atun et al. guided data collection and analysis [9, 10]. This conceptual framework was selected as fostering integration of an intervention, such as guidelines on health shops, into a given context is “both relational and complex” due to a pluralistic set of providers, diverse norms, values as well as less formal and horizontal mechanisms which shape coordination, accountability, health practice and health seeking behavior in communities or different contexts [9].

**Fig. 1:**
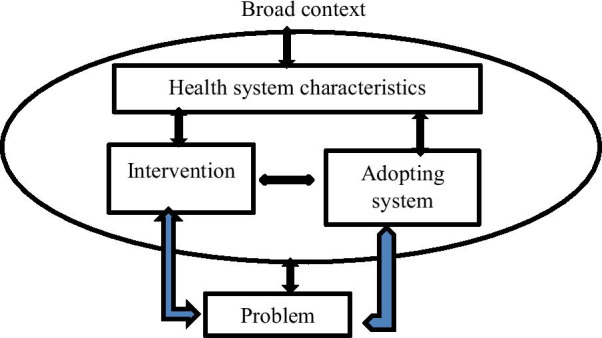
Conceptual framework for analyzing integration process (adopted from Atun et al. [10])

Atun et al. provide a systematic conceptual framework for analyzing the integration of interventions or initiatives into complex systems, such as the Zambian Health system [9]. According to this framework (Fig. 1), examining the integration process requires exploring the *nature of the problem *being addressed (e.g., lack of accredited drug shops), the *intervention *(i.e., guidelines on health shops)*, *the *adoption system* (pharmacies, shops), the *health characteristics* (i.e., staff, financing, supplies), and the *broader context* (socio-cultural factors).

### Methodology

This research project adopted a qualitative case study design which involved full participation of key stakeholders such as ZAMRA and their partners, namely the Ministry of Health and representatives from pharmacies, during the research process. This study design was selected to facilitate collection of in-depth contextual information on the acceptability and adoption of the guidelines on health shops. The study was conducted in Muchinga and the Western provinces of Zambia, the provinces where the guidelines were implemented.

The study adopted a purposive sampling method to recruit key informants in order to ensure selection of people that were representative of a cross-section of the population. The study participants were identified through the ZAMRA database. Once identified, the research team visited participants to obtain consent to participate in the study.

### Data collection methods and steps

Data were collected through documentary review and key informant interviews (KIIs) with pharmacists staff from ZAMRA, District Health Managers, health shop operators and dispensers. The KIIs provided insights on how the guidelines were developed, communicated and implemented. A total of 24 respondents were interviewed using in-depth interviews, 12 from the Western province and 12 from Muchinga province. The research team reviewed the body of literature on guidelines on operating a health shop in Zambia. Most of the health shops had been operating as drug shops before the training. Checklists were also used to ascertain the extent to which the components of the guidelines had been implemented by the health shop owners. Interviews were audio recorded and transcribed.

### Data analysis

Data were analyzed using thematic analysis. Once interviews were transcribed, authors individually read the transcripts to create codes. The preliminary codes developed by the individual authors were then discussed, applied to a small number of transcripts and re-discussed in an iterative process to develop final codes and themes. The process of developing codes was guided by the conceptual framework on the integration of health innovations into health systems [10]. This iterative process involved continuously moving between the themes and back to the data.

### Ethics

The protocol was approved by the ERES CONVERGE IRB as well as the Ethics Review Committee of the World Health Organization. Permissions were also sought from the National Health Research Authority and relevant district authorities to conduct the study. In conducting the recruitment, ZAMRA provided a list of the contact details of people that were operating health shops to the research team. These people had already provided consent to ZAMRA during the project inception to have their contact details shared with other project partners, like the research team. Once the stakeholders agreed to meet the research team, the team sought informed consent from all eligible participants before they could participate in the study. Given that the sample size was small, voluntary participation was ensured by emphasizing that participation in the study was not mandatory. Furthermore, the participants were assured that there would be no consequences should the owners of the shop refuse to take part in the study, including loss of any benefits such as possible trainings. Confidentiality was observed throughout the study period, with no disclosure of personal identifying information. All interviews were conducted in private. All information gathered was strictly secured and only authorized persons had access to sensitive information.

## Results

The results have been presented around the conceptual framework proposed by Atun et al. [10]. The section starts by describing the problem regarding lack of access to care and medicines. It then proceeds to discuss the enablers and barriers to acceptability of guidelines within the Zambian Health System. The Atun framework has been used to classify the factors that shape the acceptability of the guidelines and training, namely—intervention, adopting system, health system characterizers and broader context.

### Nature of the problem

#### Inadequate access to quality medicines

Review of the Guidelines on Operating Health Shops showed that the guidelines were implemented to address the problem of poor and inadequate access to quality medicines especially in rural areas in Zambia. Key problems that affected delivery of quality medicines included lack of skilled personnel, limited adherence to treatment protocols, dispensing drugs without prescription as well as improper use of prescription. In some cases, the medicines were not properly package and labeled***.***

### Attributes of the intervention

#### The importance of training

The requirements of operating health shops in Zambia were stipulated in ZAMRA guidelines; health shop owners were expected to follow these guidelines once registered. These guidelines covered processes on registering health shops, staff requirements, standards of health shop premises and prescriptions among other issues. Findings showed that shop owners or operators did not have knowledge on key issues around the requirements of a health shop before they were trained on the guidelines. It was reported that the training that was conducted for shop owners and dispensers created awareness on key features of a health shop among these people.

*“We have been sensitized that we should have a two-roomed store measuring 7 by 8 square meters, one as a store room and the other as where to dispense the drugs from”* (06, Male, Heath Shop Owner, Muchinga Province).

Furthermore, apart from educating operators on the infrastructure requirements needed to set up the health shop; respondents also indicated that they were trained on various family planning methods, including oral and injectable contraceptives as highlighted below.

*“We were trained on the guidelines that involve the drugs, contraceptive pills, the injectable ones and those ones which are supposed to be implanted”*

(08, Male, Health Shop Owner, Western Province).

Although they had learnt about guidelines and also family planning, they reported that the high cost of purchasing family planning products from suppliers had affected operations of health shops. Respondents reported that some contraceptives were bought at a very high price. Due to this high order price, health shop owners reported that they have to sell the drugs at higher price which was not affordable to most people in the community.

*“Family planning products ----are now very expensive because of the high order price…”*

(02, Female, Health Shop Operator, Muchinga Province).

In addition to high order price, respondents pointed out that the cost of running a health shop was high because the medicines, including contraceptives, were purchased from outside the provinces. Health shop owners purchased medicines outside the province as the drugs were not always available in the province. Ordering medicines outside the province attracted high transportation costs.

*“The barrier that affects effective stocking and operating the health shop is distance from Lusaka. You know, at retail level to order-, it is a bit challenging to travel...”*

(01, Male pharmacist, Muchinga Province).

It was suggested that there is need to come up with measures of reducing the high order price. One respondent suggested that the price of family planning could be reduced through engaging many companies in supplying family planning products.

*“The recommendation that I would make is to engage other companies to start supplying medicines such as family planning pills like Microgynon. If you look at Microgynon (at) this time it’s very expensive”*

(02, Female, Health Shop Operator, Muchinga Province).

### The guidelines are important

Most participants demonstrated good knowledge of the guidelines and were able to remember the answers to three or more questions around them. These issues which were understood by the stakeholders included legal requirements for operation, location of the shops, infrastructure requirements, security, and shop appearance and labeling. The majority of participants were able to recall and explain most of the issues they had learnt about the guidelines. Respondents noted that this was possible because the guidelines were simple for them to understand.

*“The guidelines are important and also easy to understand, and thus many of us were fine with the guidelines”* (IDI, 07, Male, Shop Owner, Operator, Muchinga Province).

One particular section of the guideline, waste management processes, was not fully appreciated by many shop operators. Some health shop operators explained that they did not fully understand how to dispose-off health waste that they generated. Thus, there is need for more training on good waste management practices.

*“On waste management; we were not told exactly how to clear waste materials and to manage waste disposal. We need guidelines on how to dispose waste (whether to burn or bury)”*

(IDI, 13, Male, Health Shop Dispenser, Western Province).

In general, several issues affected the ability of the health shop operators to implement the guidelines.

### Financial burden of implementing the guidelines

Most shop owners had financial challenges in meeting minimum standards for operating their health shops. For example, the majority of participants indicated that they did not have enough finances to make the necessary infrastructure renovations as stipulated by the guidelines. Onsite observations showed that majority of these shops were small businesses that had challenges in raising sufficient capital to comply with guidelines.

As a result of the high cost required to implement the guidelines, it was observed that a number of health shops had closed down at the time of the data collection. It was reported that the owners had decided to close down the shops in order for them to raise enough capital for infrastructure renovations.

*“The type of buildings and measurements on the building requirement they are so difficult to meet, they should relax their requirements”*

(05, Male, Health shop Dispenser, Muchinga Province).

“*Yes, they (contents of the guideline) are good, but others (guidelines) are not, as they have caused some shops to be closed”*

(IDI, 07, Male, Shop Owner, Operator, Muchinga Province).

The health shop operators understood the importance of registering their businesses with the relevant authorities. However, respondents highlighted that the registration fee and other annual fees were very high. The operators noted that this was because they were required to register with different institutions such as the local council, Zambia Medicines Regulatory Authority (ZAMRA), and Ministry of Health. Hence, most of the health shop operators had not yet registered their businesses as legally required.

*“……That is annually, and then we have to pay also for fire certificate, waste collection and they are charging K1343 (about 80 USD) for a license for a small business like this one… We talked with the ZAMRA people that if they could give us an ample time to start raising income---so that we should operate correctly”*

(IDI, 13, Male, Health Shop Dispenser, Western Province).

They thus suggested that the fees be reduced or waived to facilitate registration. Others suggested that health shops should pay one central institution only in order to reduce the financial and bureaucratic burden.

*“The other thing that can make us in the rural areas to fail [to register] is that -starting from the application, the fees [registration fees] are high. And in terms of making applications, why can't they make it easy”*

(11, Female, Health Shop owner, Western province).

Participants also observed that the registration process was even more expensive because shop owners had to travel to the capital city, Lusaka to register their business. Hence, they recommended that the registration process should be decentralized in order to reduce travel costs. One shop owner said this:

*“We ask that they bring application services of acquiring a license closer to us the people of Nakonde”*

(IDI, 06, Male, Heath Shop Owner, Muchinga Province).

Another challenge of operators in rural areas included difficulty in finding shops to rent and land to build a shop. They suggested that there is a need for councils to help them acquire spaces to enable them operate shops in rural areas.

“*Here in Nakonde the space is limited and almost all the land is owned by someone so it is difficult for us to find a structure that is meeting the requirements in the guidelines. The location really affects our business because there is no enough land and most of it is customary land so the land is limited and we just have a small piece of land which poses a challenge”*

(04, Male, Health Shop Operator, Nakonde District).

Considering the unique challenges of rural areas, it was also suggested that authorities consider removing registration fees for operators in rural areas in order to motivate people to operate health shops in these areas.

### Restricted list of drugs affecting choices

In addition, most of the participants felt that the list of drugs approved for sale by health shops was too restrictive thereby affecting profit and choice of customers. As a result, most health shops were involved in selling other products alongside approved drugs, contrary to the guidelines. Therefore, health shop owners appealed that the drug list be expanded.

*“It [health shop guidelines] helps us in that it guides us on the type of drugs to sell and not to sell. As of now we have no problems, just that the ZAMRA guidelines are too restrictive”*

(03, Male Health Shop Dispenser, Muchinga Province).

It was indicated that health shop owners’ stock oral contraceptives and male condoms because they are not allowed to administer injectable contraceptives. However, they indicated that there is a high demand for injectable contraceptives in the community. In addition, they did not stock female condoms because of low demand.

*“We are not allowed to give injections, we only give orals [contraceptive], if a patient* *wants depo provera, we need trained personnel”*

(05, Male, Health shop Dispenser, Muchinga Province).

### Health system characteristics

#### Developing governance systems

To strengthen the performance of health systems under the health shops initiative, a governance and reporting structure of a steering committee at the national level was developed. The aim of the committee was to oversee advocacy activities on the guidelines, among other issues. Further, the committee aimed to coordinate the various stakeholders involved in implementing the guidelines including the Ministry of Health, the Ministry of Local Government and ZAMRA.

### Accessibility and availability of appropriate medicines

Respondents stated that health shops provided services that were geographically accessible and additionally available to the community in instances where patients did not find the needed medicine, including family planning from public facilities. Therefore, having a health shop in the community removed the need for clients to travel long distances to the capital city, Lusaka, to buy medicines.

*“Providing the service to the community makes us feel good because sometimes you may find that the hospital does not stock certain drugs, while we are able to manage to stock, so by doing so, we are giving that service to the people who cannot manage to go to Lusaka and buy, so it makes us feel good to provide community service”*

(IDI 02, Female, Health Shop Owner, Muchinga Province).

Although access to services had improved, operators suggested that there is need for more community education on where to access various services to increase appropriate uptake of services by the public. Some respondents suggested that education should focus on the role of family planning services for young people.

*“I think sensitization yes, as long as the information is disseminated, people have the information, they know where to access what services, at what point, which steps to follow or ---and how to do it”*

(IDI, 09, Male, Pharmacist, Muchinga Province).

### Strengthened referral systems

The adoption of guidelines strengthened linkages between health shops and health facilities. Respondents reported that they now pay attention to referring complex cases. For example, respondents indicated that they were able to refer patients who had high temperature and other conditions which were beyond the capacity of health shops to manage, to the nearest hospital for advanced treatment and care.

“*Some of them bring babies, I’d check temperature, other people they come here ill, I check blood pressure then I’d write- I’d advise them maybe if the blood pressure is too high, I advise them to go to the hospital*”

(IDI 6, Male, Health Shop Dispenser, Western Province).

Some health shop operators reported that they refer women to a hospital or clinic to access injectable and other family planning methods, which they are not authorized to dispense from the health shop.

Apart from referring people for family planning services, others reported referring clients for HIV testing. Some respondents reported that they conducted HIV counseling and testing. Those clients who tested positive were immediately linked to care. At the same time, those who tested negative were offered counseling on how to avoid contracting HIV.

*“When they come to us as individuals, we advise them, we have found even people who were never tested for HIV, but according to how we talked to them, we managed to convince them, and three quarters of those we met had gone for VCT, and if found positive or negative, they would be counseled on how to live”* (IDI 13, Male, Health Shop Dispenser, Western Province).

*“They (health shops) have powers to test people for HIV and malaria. As long as their referral system is designed that those people found with malaria ‘maybe’ so at that point they do refer to the hospital”*

(IDI 15, Pharmacist, Western Province).

### The communication challenge among legal enforcers affecting integration

It was noted that while effective communication between owners of health shops and the officers from the local council is critical, communication with regulators was not always cordial. Misunderstanding between the officials from the council and the operators of health shops affected the implementation process of the guidelines. Respondents hence appealed for a friendly and understanding attitude from the legal enforcers. This is how one participant appealed to the enforcers:

*“The officers should be understanding when people have a problem and avoid using force- forced closure of the shops”* (IDI, 07, Male, Shop Owner/ Dispenser, Muchinga Province).

Respondents narrated that they would like to see increased interaction with ZAMRA to increase knowledge and ask questions about the guidelines. Compared to councils who mainly focus on enforcing payment of business operational fees and cleanliness of health shops, ZAMRA mainly focuses on monitoring and offering technical guidance to health shops regarding the need for providing quality medicines. Shop staff said that when ZAMRA visited them, they freely communicated their challenges and also got better information as opposed to using force as the case with regulators from the council. They complained that the lack of regular visits by ZAMRA had left them with some unanswered questions.

*“By working with the people from ZAMRA, you may consult with them, because some time we open these shops with little knowledge, so they should also be visiting us to educate us”*

(IDI, 05, Female, Health Dispenser, Muchinga Province).

### Adopting system

#### Improved knowledge among drug health shops

A number of the participants indicated that the training had generally helped to improve their operations and the way they served their customers. They narrated that their knowledge on drugs had improved and they were now able to give proper advice to clients on drug prescriptions. They also pointed out that the general outlook of the shops had improved their business because of the renovations that were done. One participant who had done some renovations on his shop had this to say:

*“But otherwise for the cleanliness of the shop, we also said it is also to our advantage that we will be selling in a clean environment. Even more people will be coming to your shop saying that shop is clean, and has a private room for counselling”*

(22, Health Shop Dispenser, Western Province).

Participants revealed that the community appreciated the improved services that were provided by health shops. They further stated that the community sometimes did not find medicines at the public health facilities, but when they are referred to a health shop, they received the prescribed drugs.

*“Yes, they appreciate, because they don’t find all the medicine they want from the hospital and they buy from us”*

(IDI, 03, Male, Health Shop Owner, Muchinga Province).

### Not owning the building for the health shop affecting adaptations

A majority of the health shop operators were renting the shops. Thus, it was impossible for them to make renovations to the buildings. This process was problematic as the health shop operators pay rental fees to the owners of the shops, some of whom did not see renovating the building as a priority. For example, one male participant explained and said:

*“This is making the life of some other businesses difficult. The problem is that some people are failing to get on that. They are in rented shops which don’t allow them to have whatever is stipulated in the guidelines, like having a store room, the spaces (that are) well defined”*

(IDI, 11, Female, Health Shop owner, Muchinga Province).

### Location of the health shops disadvantaging the rural areas

One of the key components in the guidelines was that health shops should be located in rural or peri-urban areas. According to shop owners, most of them decided to have their health shops in urban areas because this was more conducive for business. Being located in an urban area was more profitable as there were many people who were in formal employment compared to rural areas. Given the high cost of operating the health shops, most of them were not willing to shift their businesses to rural areas as they feared that they might have financial challenges.

“*The shop is near a train station, so it has been affected as the railway line is not operational- it affects the business very much, but if railway line is operational, you may sell K150 (8 USD) or K200 (10 USD) just like that and you knock off. If people from the community who are working come like I said, and they are those who know that you sell family planning, so they will come and buy”*

(04, Male, Health Shop Operator, Muchinga Province).

### Limited infrastructure space

While it is critical to improve health shop infrastructure, limited infrastructure space was reported as one of the bottle necks. Respondents indicated that they could not expand or convert their drug stores into health shops. They cited challenges such as installing or connecting water supplies, air conditioning and toilets, among others.

*“We cannot afford to connect water or install air cons and it’s difficult to put up proper toilets. When the inspectors inspect the shop they will not certify it due to failure of meeting these guidelines and they will not give you the license”*

(IDI, 05, Female, Health Dispenser, Muchinga Province).

### The broad context

#### Perceived improved health outcomes at community level

It was reported that some young women who face the challenge of stigmatization by health workers in health facilities prefer to access family planning services from the health shops. For instance, unmarried women who present to public facilities to request family planning supplies are sometimes denied access to products. These findings were strongly supported by the district pharmacist who had been involved in dispensing drugs in public health facilities as well as involved in supervising health shops operations.

However, some respondents reported that they provide services to all categories of people in the community. Clients who access contraceptives include single (young people) and married women among others.

*“So I would say even young people are able to access them. Like there is this thing where young ladies think if I they go to the hospital, they will start questioning me so they would rather go to the health shops and buy for themselves since these drugs are all over the shops now. Yeah, that is what I have seen but the married people mostly go to the clinics and hospitals”* (01, Male pharmacist, Muchinga province).

*“Sexually active women, and single women—we do give to them as well as married women”*

(05, Male, Health shop Dispenser, Muchinga Province).

Findings showed that improved accessibility and availability of appropriate medicines in the community, coupled with strengthened referral processes and adoption of health promotion strategies by health shops, may have contributed to improved health outcomes in the community**.** Participants indicated that patients from the community access medicine without delay in the health shops, which is a positive outcome. They stated that;

*“We are gate keepers as we quickly detect serious conditions in patients and we refer without delay to the hospital for specialized treatment. We give the opportunity to the community to get the right services and get medical attention”*

(IDI 22, Health Shop Dispenser, Western Province).

### Contextual barriers to family planning provision and uptake

Other members of the community do not access family planning from health shops because they fear being perceived or labeled as prostitutes by the community. Unmarried and single women are one group from the community who may be stigmatized by some members of the public when they are found purchasing contraceptives.

*“Some are shy because they think that people will think that if they get family planning then they are prostitutes because they are not married” *

(05, Male, Health shop Dispenser, Muchinga Province).

It was also reported that some members of the community cannot access family planning because of their religion. Some religious organizations do not allow their members to obtain family planning methods, as perhaps they think that those who do access family planning methods are sinners.

*“Because of religion, some women and other believers do not take family planning”*

(IDI, 07, Male, Shop Owner/Dispenser, Muchinga Province).

To promote access to family planning among adolescents and young people in both health facilities and health shops, it was suggested that there is a need to address the norms that hinder people from accessing the services. It was suggested that education campaigns should target both those in and out of school.

*“They don’t have information on family planning methods … Then another recommendation is that, especially the single mothers those not in marriages [from their schools]. If they could be sensitized on some of the family planning methods that would help”*

(02, Female, Health Shop Operator, Muchinga Province).

## Discussion

Health shop owners reported that they accepted the guidelines on health shops, and that training helped in improving knowledge about legal requirements around operation, infrastructure requirements, security issues, and shop appearance and labeling. Such knowledge was appreciated, as it had the potential to improve the standards of their shops and overall service delivery. Some shops, that had already improved infrastructure and the surrounding environment, reported seeing more clients seeking services, including family planning, as the shops were attractive. These findings correlate with another study in Tanzania, where it was shown that implementation of guidelines improved access to affordable, quality medicines and services in retail drug outlets in rural or peri-urban areas [2, 5, 6].

We noted that increased access to health shop services is vital in addressing local needs, especially family planning needs among adolescents. It was reported that some people preferred to access family planning services from health shops and not public health facilities due to stigma by some health workers and the community. This finding therefore supports the view that investing in community driven sexual and reproductive health services could help in improving access to family planning services among adolescents [11–15].

In addition, guideline implementation contributed to strengthening integration of different services. This happened because shops could offer more than one service such as health counselling, child health promotion, and conducting basic diagnostic tests such as rapid diagnostic testing for malaria and blood pressure checkups. Further, those with complicated cases were referred to public health facilities, indicating that the program may have contributed to a stronger referral system. This contribution is important as Miller et al. shows that health shop providers in some countries did not refer customers to other health services or provide contact details for a specialist service as they wanted to maintain clients, induce loyalty and prevent customers from fulfilling their requests and get better service elsewhere [7].

However, while health shop owners generally appreciated the guidelines, some reported that it was not easy to implement the guidelines. One of the barriers to implementing the guidelines was cost. It was reported that high registration costs led to the closure of some shops. Further, some regulatory bodies were located outside the province thereby requiring them to incur transport costs. The registration costs, coupled with the high cost of ordering family planning medicines, made some of the products very expensive. In addition, the cost of renovating health shops to adhere to expected infrastructure standards was difficult, as small business outlets with limited capital could not manage the costs. These cost issues made implementation of the guidelines difficult for some, leading to health shops, especially those in rural and peri-urban areas, being forced to close.

It was feared that the closure of shops would affect access to health products, especially family planning products. Low access might further negatively impact health outcomes, especially among adolescents, by limiting the sources or availability of reproductive health services in the community. Further, this may result in reproductive health inequalities due to limited alternative sources of family planning products [13, 16, 17].

Responsiveness includes non-clinical aspects of the health system, such as rightful expectations to be treated with dignity, being given comprehensive health information and assurance that health status will be kept confidential by health providers [13, 16, 18, 19]. Given the socio-cultural fears that adolescents have regarding accessing family planning services in public health facilities [18, 20], closure of the shops raises questions about how well the SRH needs of the adolescents, especially the needs for health information and contraceptives, are being met in communities [13]. This question is based on the findings from this study and other studies in other settings which suggest that adoption of guidelines enabled health shop owners to communicate basic instructions for medication before customers bought the medicines [7].

Closing of shops, especially in rural areas, may affect equitable access to services and the realization of universal health coverage. This assessment is based on literature, which shows that health shops have the potential to improve health coverage especially in rural areas [2, 5, 6]. To address the challenges with the implementation process, we recommend that the implementation of the guidelines should pay attention to equity through doing an equity analysis of shops when implementing the guidelines, to identify shops with low capital investment. Such an approach can help by encouraging the waiving of some registration fees for shops with relatively low business, especially those located in rural areas. It can also take the form of bringing registration services closer to the communities or districts. Reductions in operational costs may help the health shops reduce the cost of family planning products which are procured at a high cost.

While people appreciated having some regulations on the type of products provided by the shops, they noted that restrictions on provision of injectable contraception affected their ability to meet the family planning needs in the community. It was reported that injectables were the preferred family planning method among females. Thus, not allowing a health shop to provide this denied care to patients who did not want to go to health facilities. This may contribute to not using any family planning method for some. Limiting family planning options is potentially detrimental in particular for adolescents. For health services to be beneficial to young populations they should be adolescent-friendly, that is; accessible, acceptable, equitable, appropriate and effective for different youth subpopulations [10, 19, 21].

## Conclusion

The perceived benefits of guidelines for health shops at the community level, such as facilitating increased availability of key medicines and quality health services, positively influenced acceptability of the guidelines. On the other hand, the high operational and financial costs of implementing guidelines, due to multiple fees, transport costs and high costs of some family planning products, affected their uptake. Furthermore, shops being located in small spaces, lack of ownership of buildings by health shop operators and poor communication by the regulator also affected the acceptability and adoption of guidelines. Key policy recommendations include reducing the implementation costs of the guidelines by decentralizing registration of health shops, integrating the registration process for the health shop under one institution as well as providing tax waivers for pharmaceutical products being sold by the health shops to act as an incentive for health shops to operate in rural areas. Finally, it will also be important to provide training as well as refresher courses on the guidelines to staff that operate health shops.

## Data Availability

The datasets during and/or analyzed during the current study are available from the corresponding author on reasonable request.

## Abbreviations

KII: Key informant interviews
LMICs: Low- and middle-income countries
MoH: Ministry of Health
ZAMRA: Zambia Medicines Regulatory Authority
